# Supplementary material for: The exquisite specificity of human protein arginine methyltransferase 7 (PRMT7) toward Arg-X-Arg sites
Source: PLoS One. 2023 May 22;18(5):e0285812. doi: 10.1371/journal.pone.0285812 (PMC10202292; doi:10.1371/journal.pone.0285812)
Supplement: S1 Raw images — (PDF) [file pone.0285812.s003.pdf]

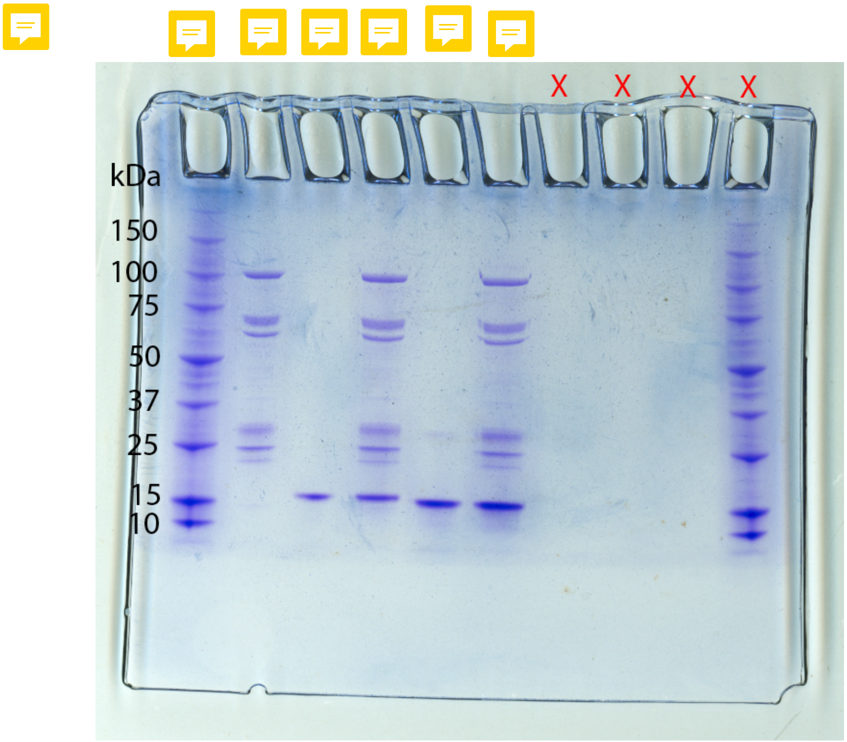
kDa
150
100
75
50
37
25
15
10
X
X
X
X

JB pg. 69
GST-HsPRMT7
→ Hs H2B
→ xe H2B
24-hr incubation
1 day exposure

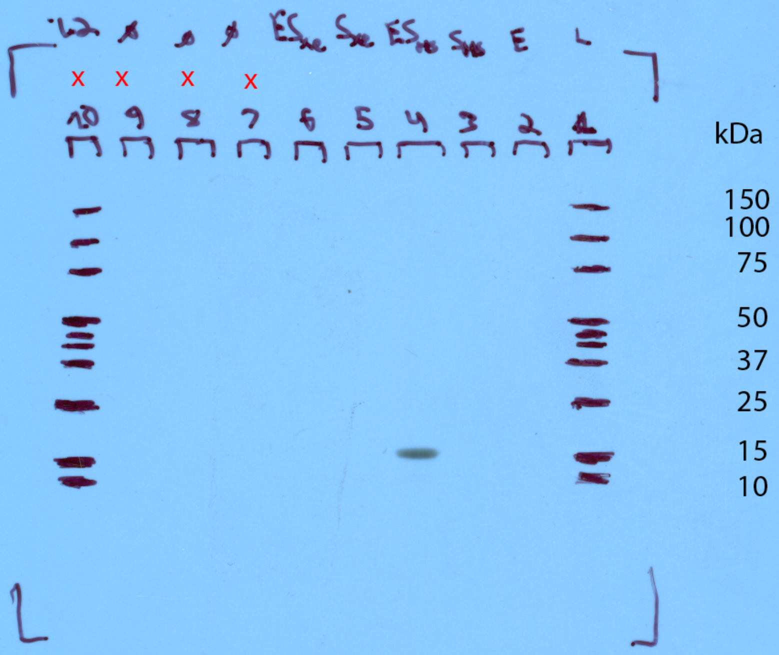

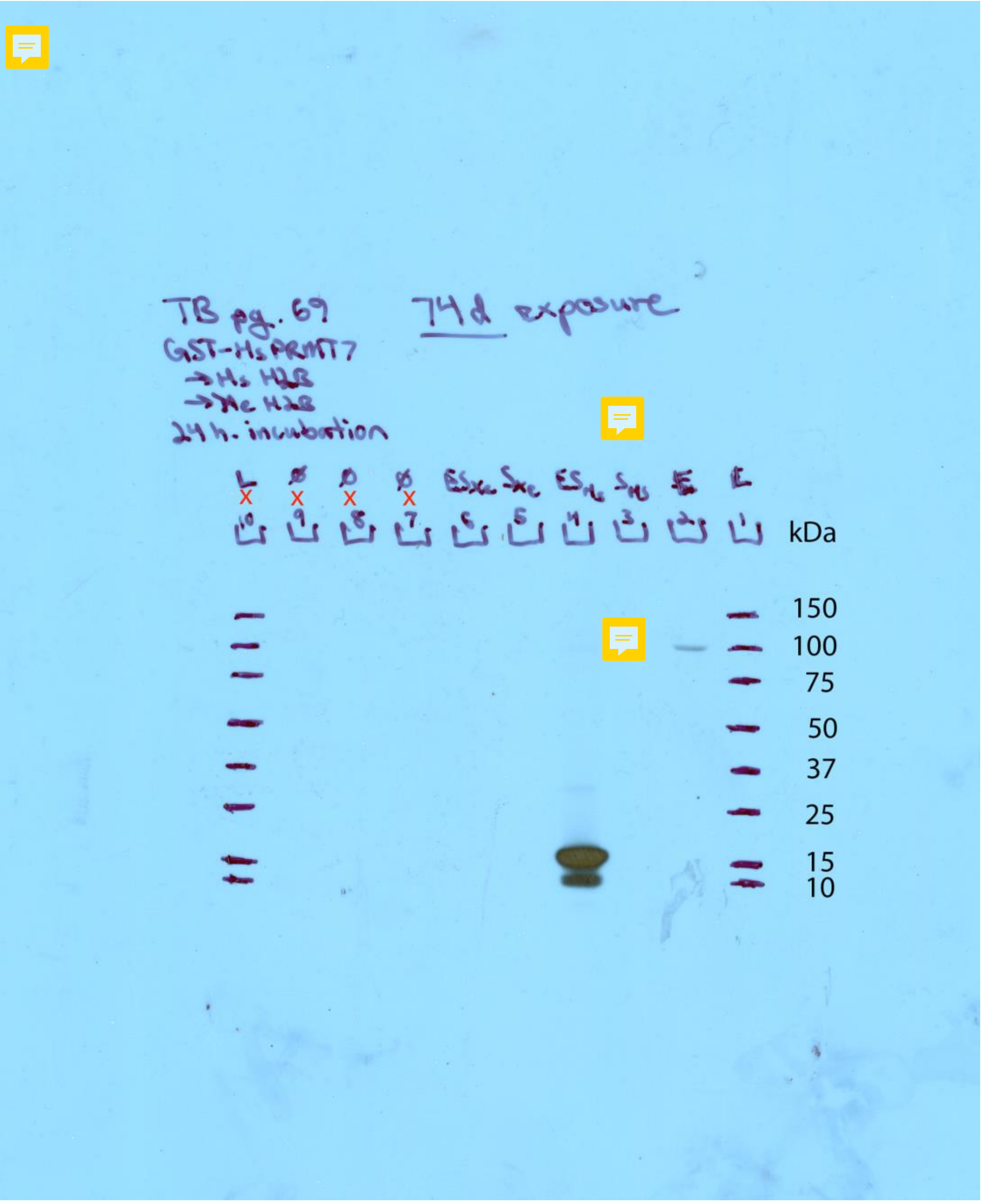

TB pg. 69
74 d exposure
GST-HsPRMT7
→Hs H2B
24 h. incubation
10
9
8
7
6
5
4
3
2
1
kDa
150
100
75
50
37
25
15
10
